# Factors Associated With 72-Hour Emergency Department Revisits at a Tertiary Care Hospital in Saudi Arabia

**DOI:** 10.7759/cureus.105741

**Published:** 2026-03-23

**Authors:** Maan Jamjoom, Baraa B Milibari, Bsaim A Altirkistani, Abdulmajeed Alharbi, Faisal Shesha, Abdulrahman Alqurashi, Hamid S Alhamid, Khalid Alansari, Faisal Boker, Ziyad Badri

**Affiliations:** 1 College of Medicine, King Saud Bin Abdulaziz University for Health Sciences, Jeddah, SAU; 2 Emergency Medicine, Ministry of National Guard-Health Affairs, Jeddah, SAU; 3 Emergency Medicine, King Abdullah International Medical Research Center, Jeddah, SAU; 4 Urology Surgery, Ministry of National Guard-Health Affairs, Jeddah, SAU; 5 Pediatrics, King Abdullah Specialized Children's Hospital, Ministry of National Guard-Health Affairs, Jeddah, SAU; 6 Emergency Medicine, East Jeddah Hospital, Jeddah, SAU; 7 Adult Neurology, King Abdullah Medical City, Makkah, SAU

**Keywords:** 72 hours, emergency department (ed), epidemiology, patient outcome, revisits

## Abstract

Introduction: The number of emergency department (ED) visits has increased locally and internationally; however, not all these visits require immediate treatment. A contributing factor to ED crowding is ED revisits. An ED revisit is defined as a patient presenting to the ED within 72 h after discharge from the previous ED visit. This study aimed to provide insights into the patterns and characteristics of ED revisits, specifically investigating the factors contributing to revisits within 72 h of discharge.

Methods: This retrospective study investigated patterns of revisits to the ED at King Abdulaziz Medical City, Jeddah, Saudi Arabia. The study included patients aged ≥14 years who returned within 72 h of discharge between January 1 and December 31, 2022.

Results: A total of 461 patients were included in this study, of which 261 (56.5%) were females. At the first ED visit, 415 (90%) of the patients were discharged directly from the ED, and only 25 (5.4%) required admission and were then discharged. However, the same patients visited the ED within 72 h, with 313 (67.9%) being discharged directly from the ED without the need for admission or transfer during the second ED visit, while 134 (29.1%) required admission and were discharged after their hospitalization. The primary cause of revisits was the recurrence of the same complaint in 199 patients (43.2%).

Conclusions: This study provides important insights into the patterns of ED revisits. The most common reasons for revisits were illness-related factors, such as the recurrence of complaints and a lack of improvement in patients’ conditions.

## Introduction

The emergency department (ED) is defined as a facility that provides assessment, diagnosis, and treatment for patients at any time [1]. Earlier reports indicated a significant increase in ED visits globally, approximately 20% during the years leading up to 2008, highlighting the growing demand for emergency services [2]. In the United States, data from the National Hospital Ambulatory Medical Care Survey showed that ED visits reached 146 million in 2021, during the COVID-19 pandemic, highlighting the substantial burden placed on emergency services during this period [3]. Despite this surge, not all visits necessitate immediate treatment. This increase in ED visits has led to substantial crowding, adversely affecting patient outcomes, increasing wait times, and placing additional strain on healthcare providers and systems [4].

In the Middle East, particularly in Saudi Arabia, information regarding frequent ED visits and patient demographics remains limited [5]. A major contributor to ED crowding is the high rate of revisits, defined as patients returning to the ED within 72 h of their initial visit [6]. Recent studies among adult ED patients in the United States have reported three-day ED revisit rates of approximately 8%, with diagnosis- and state-specific rates approaching or exceeding 10%, highlighting the clinical and healthcare burden associated with return visits [7]. Furthermore, revisits serve as crucial quality indicators of the care provided [8]. Factors contributing to these revisits include inadequate assessment or management during the initial visit, the natural progression of illnesses, and patient-related issues such as anxiety and lack of follow-up care [9,10].

ED revisits not only exacerbate crowding but can also delay treatment for other urgent cases, potentially compromising care [11]. Additionally, return visits that lead to hospitalization are associated with increased healthcare utilization and costs, further contributing to the economic burden associated with ED revisits [12]. In Saudi Arabia, a study among children with chronic diseases revealed a revisit rate of 11% [13]. Furthermore, a retrospective study reported that 32.5% of ED return visits within 72 hours were considered avoidable through improved patient education and better management during the initial visit, highlighting the need for targeted interventions [14]. These statistics highlight the pressing need for improved understanding and management of ED revisits. Therefore, this study aimed to provide insights into the patterns and characteristics of ED revisits, contributing to enhanced patient care and resource management at King Abdulaziz Medical City, Jeddah.

## Materials and methods

This retrospective observational study was conducted at the ED of King Abdulaziz Medical City, Jeddah, Saudi Arabia, a tertiary healthcare facility that provides emergency and specialized services to a large urban population. The study aimed to assess the patterns and characteristics of patients who revisited the ED within 72 hours of discharge. The study period extended from January 1 to December 31, 2022.

All patients aged 14 years and older who returned to the ED within 72 hours of their previous visit during the study period were included. Patients who revisited after more than 72 hours, those younger than 14 years, and those with incomplete or missing electronic medical records were excluded from the analysis. Patients who revisited for administrative or non-clinical purposes were also excluded. All eligible ED return visits occurring within 72 hours during the study period were included. Eligible cases were identified consecutively from the hospital’s electronic medical record (EMR) system, with exclusions applied only according to the predefined criteria.

Data were retrieved from the EMR by trained data collectors under the investigator's supervision. Extracted variables included demographic characteristics, medical history, timing of revisit, chief complaint, vital signs, pain scores, triage category, length of stay in the ED, hospitalization status, and final outcome. Patient characteristics such as age, sex, and chronic diseases (including hypertension, diabetes mellitus, and asthma) were collected and described to provide context for ED revisit patterns. These variables were not used as exclusion criteria and were reported descriptively only; no statistical adjustment or multivariable analysis was performed to control for potential confounding.

The primary outcome of this study was the frequency and cause of 72-hour ED revisits. Secondary outcomes included the rate of hospital admissions following revisits and the distribution of revisit etiologies (illness-related, system-related, and treatment-related). Data were entered, organized, and analyzed using Microsoft Excel (Microsoft Corporation, Redmond, Washington, United States) and IBM SPSS Statistics for Windows, Version 23 (Released 2015; IBM Corp., Armonk, New York, United States). Categorical variables were summarized as frequencies and percentages, while continuous variables were expressed as means with standard deviations. Because this study was primarily descriptive in nature, no inferential statistical tests or p-values were calculated. Missing data were managed using logical imputation when possible, and data consistency was verified through manual cross-checking.

Ethical approval for this study was obtained from the Institutional Review Board (IRB) of the King Abdullah International Medical Research Center (KAIMRC) in Jeddah, Saudi Arabia (IRB number: IRB/1766/23). The study was conducted in accordance with the principles outlined in the Declaration of Helsinki. All patient data were anonymized and handled confidentially, with no identifying information included in the analysis. 

## Results

A total of 461 patients were included in this study, of which 261 (56.5%) were females. The mean age was 46.6 (±20.5) years. The number of patients with no known past medical or surgical history was 151 (32.8%). Hypertension and diabetes constituted the majority of comorbidities in the study population, with 150 (32.5%) and 134 (29.1%), respectively (Table 1).

**Table 1:** Bio-demographic characteristics

Variable	Frequency (%) / Mean ± SD
Age (years)	46.6 ± 20.5
Medically and surgically free	151 (32.8)
Hypertension	150 (32.5)
Diabetes mellitus	134 (29.1)
Oncology	62 (13.4)
Dyslipidemia	58 (12.6)
Cardiac disease	50 (10.8)
Bronchial asthma	37 (8.0)
Hypothyroidism	31 (6.7)
Psychiatric disorder	28 (6.1)
Benign prostatic hyperplasia	25 (5.4)
Chronic kidney disease	23 (5.0)
Gastroesophageal reflux disease	13 (2.8)
Stroke	13 (2.8)
End-stage renal disease	11 (2.4)
Sickle cell disease	11 (2.4)
Epilepsy/seizure disorder	11 (2.4)
Biliary cholelithiasis	11 (2.4)
Osteoporosis	11 (2.4)
Liver disease	9 (2.0)
Migraine	9 (2.0)
Multiple sclerosis	7 (1.5)
Deep venous thrombosis	4 (0.9)
Recurrent supraventricular tachycardia (SVT)	4 (0.9)
Parkinson’s disease	2 (0.4)
Chronic obstructive pulmonary disease (COPD)	1 (0.2)

**Figure 1:**
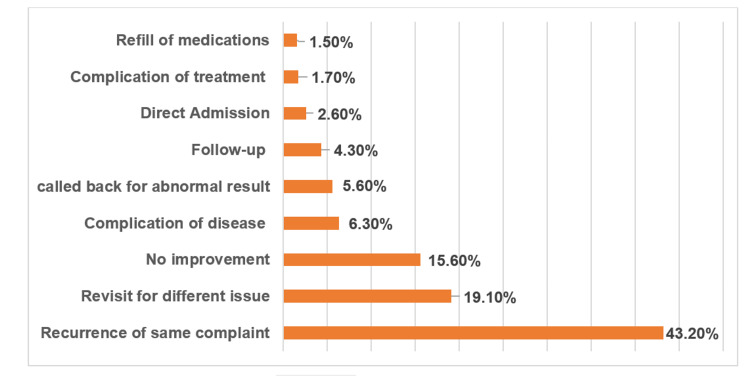
Reasons for the second ER visit within 72 hours

Additionally, 151 (32.8%) of the patients presented with abdominal pain, 66 (14.3%) had nausea, and 58 (12.6%) had vomiting. Only 34 (7.4%) had chest pain complaints upon presentation to the ED (Table 2). At the first ED visit, 415 (90%) of the patients were discharged directly from the ED, while 25 (5.4%) required admission and were then discharged home. However, the same patients visited the ED within 72 h, with 313 (67.9%) being discharged directly from the ED without the need for admission or transfer during the second visit, and 134 (29.1%) requiring admission and being discharged after their hospitalization (Table 3). The majority of revisits were illness-related, including recurrence of the same complaint in 199 patients (43.2%), lack of improvement in condition in 72 patients (15.6%), and presentation with a different medical issue in 88 patients (19.1%). System-related and treatment-related causes accounted for the remaining revisit cases (Figure 1).

**Table 2:** Chief complaints

Complaint	Frequency (%)
Abdominal pain	151 (32.8)
Nausea	66 (14.3)
Vomiting	58 (12.6)
Fever	54 (11.7)
Shortness of breath	40 (8.7)
Cough	39 (8.5)
Chest pain	34 (7.4)
Dizziness	33 (7.2)
Headache	32 (6.9)
Body pain	32 (6.9)
Back pain	23 (5.0)
Trauma	20 (4.3)

**Table 3:** Outcome, admission, and specialty (first and second visit data) *Still admitted: refers to patients who were hospitalized at the time of data collection following their second visit to the emergency department (ED).

Category / Specialty	First Visit Frequency (%)	Second Visit Frequency (%)
Discharged from ED	415 (90.0)	313 (67.9)
Discharged after admission	25 (5.4)	134 (29.1)
Discharged against medical advice	20 (4.3)	5 (1.1)
Transferred to other hospitals for admission	1 (0.2)	0 (0.0)
Deceased	0 (0.0)	7 (1.5)
Still admitted*	0 (0.0)	2 (0.4)
Admission – any department	25 (5.4)	142 (30.8)
Admitting Specialty		
Obstetrics & gynecology	6 (21.4)	40 (28.2)
Internal medicine	6 (21.4)	33 (23.2)
Cardiology	2 (7.1)	2 (1.4)
Nephrology	2 (7.1)	2 (1.4)
Hematology	2 (7.1)	4 (2.8)
Medical oncology	2 (7.1)	16 (11.3)
Vascular surgery	1 (3.6)	3 (2.1)
General surgery	1 (3.6)	20 (14.0)
Gastroenterology	1 (3.6)	3 (2.1)
Orthopedics	1 (3.6)	0 (0.0)
Adult neurology	1 (3.6)	9 (6.3)
ENT (otolaryngology)	0 (0.0)	2 (1.4)
Pulmonology	0 (0.0)	1 (0.7)
Oral & maxillofacial surgery	0 (0.0)	1 (0.7)

## Discussion

This study demonstrated that gastrointestinal symptoms, particularly abdominal pain, nausea, and vomiting, were the most frequent causes of ED revisits. Previous findings have shown that abdominal pain is one of the leading presenting complaints in adult ED visits, surpassing other common conditions such as dyspnea and chest pain. These results highlight the persistent predominance of gastrointestinal presentations as a key driver of ED utilization. Because these complaints can represent a wide spectrum of conditions ranging from mild self-limiting illness to life-threatening pathology, careful evaluation, appropriate investigations, and clear discharge instructions are essential to reduce early returns [15].

The high proportion of illness-related revisits in this study, particularly those attributed to recurrence of the same complaint and lack of improvement, reinforces observations from previous literature that unresolved symptoms are a common reason for repeat ED attendance [16]. However, given the retrospective design of this study and reliance on EMR documentation, it is not possible to distinguish whether these revisits resulted from the natural progression of disease, limitations of initial assessment or management, or patient-related factors such as expectations or access to follow-up care. Nevertheless, these findings highlight potential opportunities for optimizing initial evaluation, symptom control, and discharge instructions, including providing clearer guidance on expected symptom trajectories and indications for return to care.

Gastrointestinal and respiratory complaints, such as abdominal pain, cough, and shortness of breath, often coexist, and this overlap was observed in our cohort. Prior studies have demonstrated that integrating vital signs, particularly respiratory rate, with chief complaints during the initial ED assessment can aid in early risk stratification and identification of patients at higher risk of adverse outcomes [17]. While such approaches may support triage and prioritization of care, further studies are needed to determine their impact on diagnostic strategies, resource utilization, and ED revisits.

In the current study, most revisit patients were female, with a mean age of 46.6 years; this observation is descriptive in nature, and no inferential statistical analyses were performed to assess its statistical significance. Studies from tertiary hospitals in Saudi Arabia have reported variable associations between sex and frequent ED utilization, with some identifying female predominance and others reporting male sex as a predictor of frequent visits [18]. Gender-related differences in healthcare-seeking behavior, chronic disease burden, and symptom perception may partially contribute to this observed pattern. In addition, broader contextual and health system factors, including variations in access to care, socioeconomic conditions, and patterns of health service use, have been associated with ED use and revisit behavior in population-based studies [19]. Addressing these factors through tailored patient education and improved follow-up strategies may help enhance post-discharge adherence and potentially reduce avoidable ED revisits.

The predominance of illness-related revisits found in this analysis corresponds with broader evidence suggesting that most 72-hour returns are related to disease progression rather than inadequate initial management. Nevertheless, a small percentage of revisits were attributed to complications of pre-existing conditions or treatment-related issues. These cases emphasize the importance of adherence to evidence-based clinical guidelines, cautious prescribing practices, and comprehensive discharge counseling to minimize potentially avoidable morbidity [20].

Comorbid conditions also played a notable role in ED revisits. Hypertension and diabetes mellitus were the most prevalent chronic diseases among revisit patients, consistent with national data demonstrating a high burden of these non-communicable diseases in the Saudi population. This emphasizes the need for integrated chronic disease management programs that provide continuity of care beyond the ED. Strengthening communication between emergency physicians, primary care providers, and specialty clinics can help manage chronic illnesses more effectively and reduce reliance on emergency services [21].

Limitations and recommendations

One limitation of this study is its retrospective design, which relies heavily on documentation by emergency physicians in patients’ EMRs. These records occasionally lacked sufficient detail to fully explain the reasons for revisits, potentially leading to incomplete data and misinterpretation of findings. Additionally, this was a single-center study, which may limit the generalizability of the results to other healthcare settings with different patient populations or practices. There is also a risk of selection bias, as only patients who revisited the ED within 72 hours were included. An important additional limitation is the absence of inferential statistical analyses; therefore, the results are descriptive and cannot be used to determine the statistical significance or causal nature of the observed associations.

Future research should incorporate multicenter designs and robust statistical modeling to better identify independent predictors of ED revisits. Evaluating targeted interventions such as structured discharge education, standardized return-precaution counseling, and early follow-up pathways for high-risk patients may help reduce potentially avoidable revisits. Moreover, the use of standardized classification frameworks for revisit causes could enhance the consistency, comparability, and interpretability of future studies.

## Conclusions

This study provides valuable insights into the patterns of ED revisits in one ED in Saudi Arabia. The most common causes of revisits were illness-related factors, such as recurrence of the same complaint and lack of improvement in patients’ conditions. Awareness of these revisit patterns is important, as they may influence resource utilization, healthcare worker workload and burnout, and patient satisfaction with the care delivered.
